# Change Narratives That Elude Quantification: A Mixed-Methods Analysis of How People with Chronic Pain Perceive Pain Rehabilitation

**DOI:** 10.1155/2016/9570581

**Published:** 2016-12-14

**Authors:** Timothy H. Wideman, Alice Boom, Jennifer Dell'Elce, Kate Bergeron, Janick Fugère, Xiangying Lu, Geoff Bostick, Heather C. Lambert

**Affiliations:** ^1^School of Physical and Occupational Therapy, McGill University, Montreal, QC, Canada; ^2^Centre for Interdisciplinary Research in Rehabilitation of Greater Montreal, Montreal, QC, Canada; ^3^Faculty of Rehabilitation Medicine, University of Alberta, Edmonton, AB, Canada

## Abstract

Chronic pain negatively impacts health, well-being, and social participation. Effective rehabilitation often hinges on long-term changes in pain-related perceptions and behaviors. However, there are important gaps in understanding how patients perceive these changes. The present pilot study addresses this gap by using qualitative and quantitative methodologies to explore how patients perceive and experience changes in function, participation, and pain-related factors following a chronic pain rehabilitation program. A mixed-method design was used in which the core method was qualitative. Descriptive quantitative data was used to further characterize the sample. Semistructured interviews were conducted 1–6 months following treatment completion. Questionnaires were administered before and after treatment and at follow-up. Interview data was analyzed thematically. Participants' individual descriptive data was compared to established cut-scores and criteria for change. A major theme of personal growth emerged in the qualitative analysis. Participants also discussed the factors that facilitated personal growth and the ongoing challenges to this growth. The quantitative data revealed limited improvement on measures of pain, disability, catastrophizing, and depression. These findings suggest that, despite limited improvement on treatment-related questionnaires, patients can experience an important and enduring sense of personal growth. Clinical and theoretical implications are discussed.

## 1. Introduction

Chronic pain affects 1 in 5 adults and is a condition associated with significant suffering, disability, and social expenditure [1, 2]. Individuals living with chronic noncancer pain typically progress through a continuum of clinical management that begins with interventions to* eliminate* the pain condition and transition to treatments to* cope* with the pain condition. The gold standard approach for these latter interventions is interdisciplinary rehabilitation that focuses on education, self-management strategies, and physical conditioning [3–10]. Given the cost associated with these treatments they are commonly reserved for patients with multifaceted and treatment-resistant pain conditions. Patients entering these programs are often encouraged to make long-term changes in their pain-related behavior and perceptions. For many patients, the pervasiveness of their pain conditions means that these changes represent a challenging alteration in lifestyle.

While the literature supports interdisciplinary pain rehabilitation programs aimed at behavior change, there is a dearth of information about the processes associated with these changes. Past research in this area has primarily focused on using self-report questionnaires to shed light on how pain, psychological factors, and pain-related disability change over the course of treatment and following discharge [11–15]. These findings have been valuable in helping develop and test prognostic models of recovery and in helping clinicians monitor treatment progress. However, these data do not necessarily illuminate the idiosyncratic processes involved in behavior change. Moreover, self-report questionnaires are highly standardized and are designed to address a relatively narrow scope of factors. On the other hand, lifestyle changes associated with chronic pain rehabilitation have the potential to influence a broad range of factors and to shape perceptions in a subjective fashion. It is likely that traditional pain-related questionnaires only capture a subset of the factors influencing this recovery process. Given the narrow scope of self-report measures, there is a need for more comprehensive accounts of how people living with chronic pain perceive the occurrence and maintenance of treatment-related change.

Qualitative methodology may help shed further light on these processes and complement more traditional quantitative measures. Qualitative methods enable researchers to gain a broader understanding of human experiences, attitudes, and processes through an emphasis on participant perceptions [16–18]. Past research has highlighted the added value of this methodology in understanding the subjective experience of pain [19–22]. A recent randomized controlled trial found a concordance rate of less than 50% between quantitative questionnaire and qualitative interview data [23]. This finding indicates that qualitative methodology has the potential to address aspects of the pain experience that may not be readily captured by quantitative measures. It also suggests that mixed-methodologies that combine qualitative and quantitative assessments may provide a more comprehensive understanding of pain-related factors.

To date, only a small number of studies have used a mixed-methods approach to evaluate patients' experiences with and responses to pain rehabilitation programs. Previous studies in this area have often focused on perceptions relating to specific outcomes (e.g., activities of daily living) and/or discrete measures (pain-related questionnaires) [24–27]. Better understanding patient perceptions from a more broad qualitative and quantitative perspective has the potential to advance our understanding of the different facilitators and barriers to treatment and to improve clinical management.

The overarching aim of the present study was to address this need by using qualitative and quantitative methodologies to determine how patients perceive and experience changes in function, participation, and pain-related factors following a chronic pain rehabilitation program. Study participants completed semistructured qualitative interviews to address their perceptions of change. Self-report questionnaires were used to assess treatment-related change in levels of pain, disability, and psychological factors.

## 2. Methods

### 2.1. Research Design

This study used a mixed-method design that was guided by the philosophical paradigm of pragmatism, which prioritizes the generation of socially useful knowledge when using and integrating qualitative and quantitative methodologies [28, 29]. The core method in this study is qualitative and was used to provide a narrative view on change associated with treatment. The qualitative methods were based on the interpretive description framework, which aims to generate an understanding of clinical phenomena that can directly inform clinical practice [30, 31]. In this study, the quantitative data is secondary and provides a numeric, descriptive representation of treatment-related change to complement the qualitative data. The quantitative data in this study is not used to make any statistical inferences about a larger population but rather is presented to better characterize the clinical presentation of individual participants.

### 2.2. Participants

All patients that had previously completed the chronic pain management treatment program at a Montreal-based rehabilitation center within 1 to 6 months were eligible to participate in the study. Participants were eligible for the treatment program if they had a musculoskeletal chronic pain condition that was associated with physical impairments and disability and had no medical contraindications to exercise. All patients in this program received interdisciplinary (physical therapy, occupational therapy, psychology, and kinesiology) interventions focusing on pain education, psychological counselling, and progressive engagement in exercise. The overarching goal of treatment was to encourage positive coping behavior, activity engagement, and participation in social activities. Patients received treatment in group and/or individual settings that commonly lasted between 3 and 6 months.

Eligible individuals were recruited using purposive sampling. Consistent with the approach used in qualitative research, the goal of sampling was to maximize heterogeneity within the sample in order to obtain the richest possible data. Purposive sampling allows the researcher to select a range of patients with characteristics relevant to the phenomenon of interest [16, 32, 33]. For this study, the sample includes a range of ages and time since treatment completion.

### 2.3. Procedure

Potential participants were contacted by telephone by a research assistant. Interested individuals were informed of study details and sent a recruitment packet that included a consent form and four self-report questionnaires. Participants completed and returned the recruitment packet and were scheduled for a telephone interview. In consenting to participate, participants authorized the research team to consult their medical charts which included results on the same four questionnaires that were completed at the beginning and end of their treatment program.

Telephone interviews were conducted by 3 researchers (Alice Boom, Kate Bergeron, and Janick Fugère) who had received training on qualitative interviewing. Interviewers were not affiliated in any way with participants' previous or future treatment. To improve the trustworthiness of the content of the interviews and as a backup in the event of audio recording difficulties, two researchers participated in each phone call. One researcher served as a primary interviewer, and the other served as a listener to note down interview content and to provide additional prompts to guide the interview. Researchers proficient in French conducted interviews in French, while those proficient in English conducted English interviews. Note-takers were bilingual. All interviewers and note-takers met on a regular basis to reconcile any differences in the prompts given to the participants and to improve the consistency of the interview administration.

Audio recordings were transcribed verbatim and verified for accuracy against the recordings. Transcripts were anonymized by substituting identifying information with generic nouns, and the identity of each interviewee was linked to study data (transcripts and questionnaires) by an identity code.

#### 2.3.1. Sample Size and Data Saturation

Qualitative methods were the foundation of the study and therefore the sample size was determined accordingly. Qualitative studies typically use the concept of saturation to decide when enough data has been collected. Data saturation is achieved as the point at which additional interview data no longer contributes new information to the study [34, 35]. In the present study, once data saturation was suspected (i.e., no new themes emerged in interviews), a final interview was conducted. When no new information was obtained from this interview, recruitment was halted.

This project was approved by the research ethics board of the Centre for Interdisciplinary Research in Rehabilitation of Greater Montreal and informed consent was obtained from all participants. Since the project was conducted at a single site, participant demographics have been reported in summary to avoid the possibility of identification.

### 2.4. Qualitative Interview Guide

A semistructured interview guide was developed to collect information regarding how patients perceived changes in lifestyle, function, and social integration following completion of an interdisciplinary chronic pain program. The main interview question was “how has the chronic pain self-management program influenced your day-to-day functioning and participation today?” Additional probing questions addressed participants' initial expectations for change, perceptions of change during treatment, and the perceived posttreatment implications on their lifestyle, function, and social integration. The interview guide is shown as follows.

*Overarching Question: How Has the Chronic Pain Self-Management Program Influenced Your Day-to-Day Functioning and Participation Today?*

*Probing Questions*

Initial expectations of the program and whether or not these were met
Most and least helpful components of the program
Tools or skills maintained (or not) since program completion
Facilitators or obstacles to maintaining program tools
The impact of interacting with others within the group stream (if applicable)
Quality and impact of relationship with treating professionals
Overall positive or negative impacts of the program on functioning and relationships

### 2.5. Self-Report Questionnaires

Pain severity was measured via the pain severity subscale of the West Haven-Yale Multidimensional Pain Inventory (MPI) [36]. The pain severity subscale is comprised of three items that range from 0 to 6, in which higher ratings indicate more severe pain. Total scores on this subscale are calculated by averaging the three ratings. Items of the MPI are shown to display good convergent and discriminant validity and internal consistency (*α* = 0.70–0.90) [36]. Previous research has used a 30% reduction in pain severity ratings as an indicator of clinically meaningful reduction [37–39].

The Pain Disability Index (PDI) was used to evaluate levels of perceived disability across seven different life domains. The PDI uses a scale from 0 to 10, in which greater scores indicate more severe pain-related disability [40]. The PDI is a commonly used scale and has been shown to be internally reliable (ICC = 0.86) and valid for discriminating between low and high levels of disability [40–42]. Previous research has used a reduction of 9.5 points on the Pain Disability Inventory as an indicator of clinically meaningful change [43–45].

The Pain Catastrophizing Scale (PCS) is a commonly used 13-item questionnaire that was used to measure levels of pain catastrophizing [46]. Pain catastrophizing is defined as an overly negative cognitive disposition towards pain that is characterized by a magnified threat value, ruminating thoughts and perceived helplessness. Each item asks patients to rate their thoughts and perceptions associated with pain on a 0 to 4 scale, in which higher ratings indicate greater levels of catastrophizing. The PCS demonstrates good test-retest reliability (*r* = .70 to .75) and validity [47–49]. Previous research has used a cut-score of 20 on the PCS to define posttreatment recovery in levels of pain catastrophizing [38, 39, 50].

The Patient Health Questionnaire (PHQ-9) was used to measure levels of depressive symptoms. The PHQ-9 is a nine-item questionnaire that evaluates depressive symptoms over the previous two-week period. The frequencies of these symptoms are ranked on a 0 to 3 scale, in which higher scores indicate more severe symptoms [51]. Internal consistency and test-retest reliability were found to be excellent [51, 52]. The PHQ-9 was also found to have good criterion and construct validity [51, 52]. Previous research on the PHQ-9 has used a cut-score of 10 as an indicator of clinically significant levels of depressive [51, 53–55].

### 2.6. Data Analysis

#### 2.6.1. Qualitative Analysis

Interviews were analyzed using thematic analysis, a process involving the identification and naming of patterns of meaning that emerge from the interview transcripts and that relate to the research questions (i.e., coding) [34, 35, 56]. To ensure consistency across team members, the first transcript was coded concurrently by all researchers. A second transcript was then coded independently by each of the researchers, and the coding was examined to ensure that the researchers were identifying the same themes in the data. Where there was disagreement, the group discussed the codes theme in question until a consensus was reached regarding its viability. Three subsequent transcripts were coded by pairs of researchers until it was apparent that the interviews were being coded similarly. Independent coding followed for the remaining transcripts, with regular discussion for confirmation of the codes. This system ensured a consistent method for coding, which strengthened the trustworthiness and rigour of the analysis.

Throughout the coding process, codes were clustered according to their content, creating higher-order themes [18]. Definitions for the various themes that emerged during the qualitative interviews were generated [35]. As is standard in qualitative methods, data collection and analysis were concurrent. This ongoing analysis process allowed emerging categories and concepts to be further explored in subsequent interviews [57]. It also allowed the themes to be tested against incoming interview data and adapted to reflect any newly emerging themes and relationships [18], which in return shaped subsequent interviews. The entire analysis process, including discussion about themes, definition of constructs, and emergent ideas by members of the research team, was documented to create an audit trail. The qualitative software program NVivo 10 (QSR International, Melbourne, 2012) was used to organize and facilitate the coding process.

#### 2.6.2. Quantitative Analysis

Total scores for all self-report questionnaires were calculated in a manner consistent with previous research. Scores for each participant were evaluated in relation to previously validated cut-scores and/or criteria for clinically meaningful change. Where indicated, differences between pretreatment and follow-up scores were used to evaluate criteria for clinically meaningful change.

## 3. Results

Twenty-four (*n* = 24) potential participants agreed to be contacted by the researchers to explore participation in the study. Of these, 5 were unreachable by phone, and 5 declined to participate. Reasons for not participating included experiencing too much pain, lack of time, and being concerned about confidentiality. Fourteen (*n* = 14) individuals gave their consent and completed the phone interview. One participant (006) experienced considerable difficulty responding to questions addressed in the interview due to difficulty focusing on questions. This individual persistently discussed tangential themes that were unrelated to the research question. The research team met to discuss findings from this interview and concluded that there was no information that related to the present study. The interview was therefore excluded from the study. One participant (Participant 011) dropped out of the pain rehabilitation program early and did not complete the postprogram questionnaires or return follow-up questionnaires. However, she did participate in the interview. The team discussed this participant and decided to include her in the qualitative analysis despite this missing data as it was an important opportunity to integrate the perspective of a patient that may have been unsatisfied with the treatment program.

The average interview time was 47 minutes, and a total of 610 minutes of interview data were collected. Data saturation was reached after 12 interviews; an additional interview was completed to confirm that no new major themes would emerge. Thus, the final sample size was 13, which is considered sufficient for most qualitative studies as it permits intimacy with the data and a more thorough analysis [58] and which broadly aligns with the sample size in other mixed-method research in this area [59–63].

Eleven of the participants (*n* = 11) were female and two were male; their ages ranged from 32 to 71 years (median = 47). Pain duration ranged from 1 to 43 years, with a median of 8 years. Eleven participants (*n* = 11) described themselves as presently not working. Participants' self-reported their pain diagnoses as fibromyalgia (*N* = 7), nonspecific back pain (*N* = 3), degenerative disc disease (*N* = 2), and disc herniation (*N* = 1).

### 3.1. Qualitative Data

Within the interview data, common themes emerged from the participants' personal experiences. The following section outlines the findings of the qualitative analysis, including the themes generated, their definitions, and illustrative quotes.  Table 1 includes all themes, subthemes, and definitions. The major themes were* personal growth*,* factors affecting personal growth,* and* ongoing challenges*. For clarity, filler words such as “um” were removed from quotes. The quotes from interviews in French were freely translated by an author proficient in French and were then checked by a second author.

#### 3.1.1. Personal Growth

A common theme discussed by many of the participants was a sense that they had undergone personal growth during the program. Personal growth was defined as the perceived positive development in the domains of acceptance, resilience and capacity, motivation to engage in meaningful activity, and/or self-worth. For example, one individual stated: “When you get [into the program], you come out of it and it's sure you've changed” (INT002), and another described that “by the end of it [the program], I felt like I got my life back” (INT001). The theme of personal growth was derived from the following subthemes that emerged from the data: acceptance, resilience and perceived capacity, motivation for meaningful activities, and self-worth. Each subtheme is described below and supportive quotes are provided.

Participants described that the program helped them to accept their condition. One participant stated: “I see a way out now, […] I know my life will never be as it was, but I know I'm going to live it” (INT002). In addition, some participants highlighted accepting limitations associated with their pain condition: “before, I was always upset that I can't do this or that, even if I want, but now I know that for me, there is a certain limit […] I don't get so much frustrated with that” (INT007).

Participants also described an increase in perceived capacity and resilience. One participant experienced a number of physical improvements: “I'm able to stand up straighter; my core is still weak so I still use the walker, but aside from that, I'm a lot stronger physically” (INT009). For another participant, the growth fostered by the program had profound impacts on their mental health and coping skills: “I was dealing with so much, you know, abuse, thoughts of suicide, depression, all of these things [which are] pretty much gone and then the fact that I had to deal with some pretty stressful [times] but I came through it pretty unscathed. And I know that's definitely due to being able to manage my pain better” (INT001).

A number of participants described increased motivation for meaningful activity following the program. One participant said that “[t]he consultations with the psychologist were the little push that was missing for me to get engaged in volunteering, it was something I really wanted to do and before I was restricting myself from doing it” (INT004). Another participant described that they are currently seeking a new hobby due to the positive impacts it would have on their life: “The one I looked up maybe taking painting, or… Something that would be able to be a distraction. You know? Something that would help me get out a little more, plus help take your mind off the pain […] instead of just shutting myself in all the time” (INT012).

Finally, many participants showed increased self-worth following the program. For example: “Well for me [one of the positive changes] was to take more time for myself and really be a little bit more selfish to myself. Not giving too much to the others around me. Although I love my family […] when I can't go out of the house, I can't step out. I tell them no, I can't be there” (INT011). Indeed, learning to place importance on one's needs and communicate them to others was a significant change for some participants: “The aspect worked on with my partner was to try to learn how to better communicate to him how I was feeling without feeling guilty in saying it to him” (INT004).

Despite the consistent theme of personal growth across the sample, participants varied in the degree to which they supported the various subthemes.  Table 2 aims to help the reader visualize this variance by highlighting which participant endorsed which subtheme of personal growth. All participants were able to identify an increase in resilience and perceived capacity following the program. However, the 3 other themes within personal growth were represented to varying extents. Amongst the four themes in personal growth, acceptance, and self-worth are the least mentioned amongst all participants. Participants 001, 002, 004, and 005 were all fully represented under all four categories of personal growth. In contrast, Participant 003 was represented in two of the four categories, and Participant 006 was only represented in one of the four categories. The sample therefore represents a continuum, with some participants demonstrating the majority of the themes in the model, while others are underrepresented in key areas.

#### 3.1.2. Factors Supporting Personal Growth

Participants identified a number of factors that supported personal growth, including the mindset entering the program, perceived environment of treatment, and experiential learning. Each subtheme is described below and supportive quotes are provided.

Many participants noted that having a positive mindset entering the program helped to facilitate positive change. Several participants identified the motivation to change as helpful to their improvement throughout and after the program: “The motivation, the will to improve […]. When you have enough will to change, well you do it, that overtakes laziness” (INT004). Her statement is well illustrated in the accomplishments she described later in the interview, namely, adapting her exercise routine and incorporating volunteering into her schedule. Three participants also came into the program with a strong desire to learn. “[My expectations were] to understand, to know what my illness is. Am I going to get through it?” (INT002). This desire to learn brought this participant to understand her capabilities, which restored hope for moving forward: “they made me see that there was a way out for me […] they made me understand that I would never be the same, but that there was still hope that I would return, that my capacities would return” (INT002). Finally, one participant highlighted the importance of having a positive outlook for getting the most out of the program, “I try to find maximum use for myself and to look at it positive, because if you will start to concentrate [on] what is not good [in the program], what is not useful, then you always get frustrated” (INT007). These personal characteristics may have promoted participants engagement with program activities and thereby influenced their development and growth through the program.

Another factor described by participants as facilitating personal growth was the perceived treatment environment. Several participants stressed the importance of being surrounded by supportive professionals: “they were there to support and help me, that's what did me the most good in the entire program. It's the listening and the support that these people offer us” (INT004). Some participants felt validated by the professionals understanding of their pain conditions: “it was like them confirming that the pain is real […], like not feeling that I'm crazy or something” (INT011). For this participant, the validation from the professionals may have allowed her to normalize her pain experience: “I feel that even if I'm living with pain, I'm still a normal person. […] There's millions of people all over that suffer pain, maybe as much as me, or sometimes more sometimes less” (INT011). Finally, participants who underwent group treatment sessions also reported the feeling of not being alone in their pain experience: “even though the other participants in the group did not have the same pain that I did in the same location, they were all suffering from chronic pain, so there was some understanding as to what I was going through, so I didn't feel so isolated” (INT009).

One key factor impacting participants' perceptions of their own capabilities and growth was the opportunity to engage in experiential learning. Although many participants described that the exercise program was very difficult for them, going through that challenge and succeeding increased participants' perceptions of their own capacity. For one participant: “[my expectations] changed when I could see the difference in how much weight I could lift and how long I could walk, how far I could walk. And once I started seeing that, I've started becoming a lot more confident but also realistic in things I could fix and things I just have to deal with” (INT001). The feedback provided by the therapists was seen as a facilitator to reflecting about one's own progress: “I went every day and things started to happen, I didn't even realize they were happening until they were pointed out to me” (INT009).

Aside from just physical improvements, participants made gains through implementation of new strategies acquired from the education component of the treatment program. Many participants stated that they were initially skeptical about the usefulness of the program material. However, after trying some of the strategies, participants saw through experience that such strategies were helpful “[one thing I learned from the program was] how to get the proper sleeping material, like, what you need to have to help you have a good night's sleep so you wake up in the morning and you're not so stiff […] [now] because I'm not straining myself as much, my sleep is less interrupted” (INT009). In turn, seeing the helpfulness of these strategies promoted their continued use in this individual: “Interviewer: What has helped you to maintain these tools? Participant: The results […] when I do them I don't feel as much pain […] I don't have the strain in my back that I would normally have” (INT009).

One of the characteristics of the program that was highlighted by participants as being the most helpful was the provision or personal/practical tools: “It was practical […] It was real life, without any theory […], it was applied theory and showed with real objects” (INT008). In addition, the applicability of the program material to the participants improved their confidence in the professionals. One participant stated that “their way of showing us that they truly wanted to help us and that they adapt their solutions to our situation are all factors that give us confidence in them” (INT004).

This same individual was one of many participants who mentioned that learning practical and easily applied strategies helped them to better manage their day-to-day life. For this individual, these strategies were related to her work: “[the program] help me, amongst other things, to manage my […] work, to make it more of a habit to listen to myself when I need a break” (INT004).

#### 3.1.3. Ongoing Challenges to Maintaining Personal Growth

Despite having experienced many positive changes during and following the program, participants also reported ongoing challenges. For instance, some participants reported ongoing challenges in relation to acceptance of their pain condition. As stated by one individual, “I haven't really accepted it […] It's just like sometimes I feel like saying, you know, ‘I shouldn't have to accept it.' I'd like to just get rid of it” (INT012). Another participant reports “[I] live and accept [my condition], accepting it more or less but I still don't have the choice to live with it” (INT010). This data suggests that acceptance is a process, with some individuals at a stage in which they have accepted their situation and condition more than others. Similarly, participants reported ongoing challenges associated with maintaining program related skills and strategies in the face of everyday challenges such as weather, habits, and the pain experience itself. For example, one participant stated that “The cold and the humidity [make my condition worse]” (INT011). Additionally, “laziness and old habits are the biggest challenges [to maintain tools]” (INT004).

### 3.2. Quantitative Data

Table 3 shows all quantitative data and the relationship to previously established cut-scores and/or criteria for clinically meaningful change; participant scores on each questionnaire are addressed below.

#### 3.2.1. Pain Severity

Three participants in the study sample experienced greater than 30% reduction in levels of pain severity from pretreatment to follow-up (Participants 001, 005, and 010). Of the nine participants that did not meet this threshold over the same time frame, three experienced increases in their levels of pain (Participants 003, 008, and 014).

#### 3.2.2. Pain Disability

Three participants experienced clinically meaningful reductions in disability from pretreatment to follow-up (Participants 001, 002 and 005). Of the nine participants that did not meet this threshold over the same time frame, five participants experienced increases in their levels of disability (Participants 003, 004, 008, 009, and 014).

#### 3.2.3. Pain Catastrophizing

All participants except one (004) started treatment above the established threshold for this measure (i.e., higher than 20). At follow-up, five participants had levels of pain catastrophizing below the threshold (Participants 001, 004, 005, 007, and 009). Of the seven participants that did not meet this threshold, three had experienced increases in levels of pain catastrophizing from pretreatment to follow-up (Participants 003, 010, and 014).

#### 3.2.4. Depressive Symptoms

Prior to treatment, all participants with data for this variable scored at or above the established cut-score for major depressive disorder (i.e., ≥10). At follow-up, six participants scored below this threshold (Participants 001, 004, 005, 007, 009, and 013). Of the six participants that scored above this threshold at follow-up, three participants experienced increases in their levels of depression over this time frame (Participants 003, 008, and 014).

## 4. Discussion

This study reveals an interesting pattern of findings in which quantitative data show minimal treatment-related improvement, while qualitative data show important improvement during and following treatment in areas not commonly assessed in clinical settings. The quantitative data show that the majority of participants at follow-up did not achieve clinically meaningful improvements across measures of pain, disability, catastrophizing, and depression. Despite these apparent modest treatment effects, data from the qualitative interviews reveals that participants experienced personal growth that was supported by treatment- and patient-related factors. The qualitative interviews also point to treatment-related processes through which personal growth and recovery may be facilitated. These findings add to the limited mixed-methods literature on patient perceptions of pain rehabilitation and have important implications for future research, clinical practice, and theoretical models.

The study findings suggest a general discordance between quantitative and qualitative results. This discordance was particularly evident when certain participant quotes are contrasted to quantitative findings. For instance, Participant 002 stated “When you get [into the program], you come out of it and it's sure you've changed.” Interestingly, the questionnaire-based assessment for this participant paints a contradictory picture, in which this participant does not achieve meaningful changes on three of the four questionnaires. Similarly, another participant (INT004) reported personal development in all of the identified subthemes (acceptance, resilience, motivation, and self-worth), but there was considerable variance across questionnaire data; this participant's disability levels became more severe and pain levels did not improve, while psychological factors improved. On the other hand, two participants (Participant 001 and Participant 005) reported personal development in all themes and showed improvements across all measures. In general, it appears that across participants there was no clear pattern between questionnaire findings and interview content. This observed discordance is broadly consistent with the limited mixed-method research in this area. Within the context of pain, Dudgeon et al. showed that pain narratives yielded contrasting descriptions of pain qualities when compared to adjective ratings from the McGill Pain Questionnaire [64]. Research from outside the field of pain suggests that interview and questionnaire assessments yield divergent data, in part due to variance in context (interpersonal versus private) and scope (broad versus specific) [65]. It is noteworthy that, despite careful attention to a neutral line of questioning, participants chose to focus on positive rather than negative aspects of their recovery and that the theme of personal growth is one that developed organically through each interview. These findings are consistent with other research that specifically focuses on the positive experiences and perceptions of people living with pain [66].

The choice to focus interview responses on positive factors, despite apparent limitations from questionnaire responses, may stem in part from participants' integration of themes related to acceptance and resilience. One participant quote helps illustrate this interpretation of the findings: “I try to find maximum use for myself and to look at it positive, because if you will start to concentrate [on] what is not good [in the program], what is not useful, then you always get frustrated” (INT007). Here the participant clearly alludes to challenges but emphasizes the importance of choosing to focus on positive factors. This is largely consistent with the theme of acceptance that emerged throughout the interviews, in which participants emphasize learning to live with their limitations. In line with this interpretation, past research has shown that acceptance can reinforce an orientation towards the rewarding aspects of life [66–69]. Similarly, many participants described increased confidence in their ability to cope with challenges. These findings are consistent with a pain-related model of resilience, which focuses on the ability to return to homeostasis after stressful pain-related experiences and continued engagement in meaningful activities [68]. Consistent with this interpretation, it is possible that both the qualitative and quantitative data represent accurate aspects of the rehabilitation process in that limitations are still present (as shown in the quantitative data) but are deemphasized due to a new, more positive outlook (as shown in the qualitative data).

Our findings that individuals with chronic pain can simultaneously experience high levels of negative pain-related factors, such as pain, disability, and psychological risk factors, while simultaneously taking steps towards personal growth have important links to models of pain-related disability. The Fear Avoidance Model of pain is a leading model of pain-related disability that exclusively focuses on negative psychological and pain-related factors [70, 71]. Recent research has called for a more comprehensive framework of pain-related disability that incorporates both negative and positive factors related to recovery [72]. The present findings are consistent with this call and a growing body of literature that suggests that positive and negative pain-related factors are not two sides of the same coin but rather have independent variance [72, 73]. Future research will need to build on these findings by using both quantitative and qualitative methodologies to more fully integrate both positive and negative factors within emerging conceptualizations of pain-related disability.

The theme of engaging in meaningful activity was also a key factor related to personal growth and has important relevance for exploring the relationship between pain and physical activity. Poor adherence to activity-based pain rehabilitation programs remains an important barrier to improving chronic pain outcomes, with estimated adherence rates as low as 30% [74–77]. Research in the pain literature that addresses poor treatment adherence has focused on identifying correlating factors and has identified barriers such as low levels of baseline physical activity, poor social support, and depression [75]. By contrast, there has been little research investigating how to mediate these barriers, leaving clinicians with little direction on how to resolve this important issue. The bulk of the literature focusing on increasing physical activity amongst people with chronic pain conditions focuses on assessing the duration and intensity of activity completed through self-report questionnaires or objective measures [78–81]. It is possible that also evaluating the perceived meaning that patients associate with physical activity would help shed light on additional strategies to increase adherence. This is broadly consistent with previous findings which show that patients with chronic pain believe that pursuing personal interests promotes physical and mental health [82] and treatment programs that are perceived as fun and stimulating show greater levels of participation [83]. Indeed, considering patient preferences and individualizing exercise has been incorporated into the clinical practice guidelines for back pain [84]. Future research should further emphasize assessment strategies that capture activity duration and intensity as well as meaning and interest associated with physical activities. A mixed-methods approach is particularly well suited for this goal, as it could combine an interview about how patients perceive treatment-related activity, with more traditional self-report and objective measures. This strategy may help shed light on further treatment strategies to improve the longstanding challenge of adherence to activity-based interventions.

Our findings suggest that personal growth was facilitated by the safe, validating, and supportive environment of the program. This is an important finding as it is well documented that individuals with chronic pain often report feeling isolated and misunderstood by family, friends, and even healthcare practitioners [85–89]. In addition, sharing one's pain experience with others is often perceived as leading to adverse social consequences [64, 90–92], and making comparisons to others can have negative effects on self-esteem [93, 94]. However, in the present study, exposure to other patients in the program appeared to have a positive and normalizing effect on the perceived capacity of our sample and many described gaining a more encouraging perspective on their condition and an overall increase in self-worth. It is possible that this discrepancy with the previous literature is due in part to participants not making potentially demoralizing comparisons to pain-free individuals, but rather relating their experiences to other patients with similar pain-related limitations, which had a normalizing effect. Participants also highlighted that the positive perception of the treatment context was instrumental in helping them go beyond perceived limitations and experiment with new activities and practical tools. The theme of validation, safety, and support that emerged in the present sample emphasizes the importance of considering how patients perceive the treatment environment and context when developing chronic pain interventions and taking measures to ensure that it is conducive to personal growth throughout and following treatment. Similarly, past research has shown that individuals with chronic pain are more inclined to continue to engage in work-related activities if they perceive their employers to be understanding and supportive [95].

Experiential learning was another key factor supporting personal growth. Participants specifically highlighted the unique value of combining activity engagement with facilitative self-reflection as a means of learning. One participant quote helps illustrate this point: “I went every day and things started to happen, I didn't even realize they were happening until they were pointed out to me” (INT009). The participant implies that learning would not have occurred without both of these components. Both the patient and therapist likely contribute to this form of learning. Our results highlighted the importance of participant mindset at the beginning of the program, which likely creates openness to this form of learning. Our findings also suggest that specific feedback from therapists facilitates this process.

One strategy for further enhancing therapeutic feedback within clinical settings is integrating formal assessment strategies to specifically address positive factors. Previous research suggests that clinical assessment can be a useful means of providing patients with feedback regarding their progress [96–99] and that increased awareness of successful performance increases motivation and confidence [83, 100]. However, our results suggest that using traditional pain-related questionnaires on their own might not be sufficient for communicating this feedback to patients. As personal growth seemed to occur in many different contexts and in many different ways within the chronic pain self-management program, using narrative interviewing may be a viable clinical strategy available to all healthcare professionals specializing in chronic pain rehabilitation. Past research has highlighted the value of qualitative interviews in assessing patients evolving expectations over the course of treatment [101]. In addition to qualitative interviewing, the incorporation of measures of positive psychological factors, such as resilience and acceptance, may be a complementary strategy to quantitatively capture the concept of personal growth. Together, these assessment strategies may be able to further highlight aspects of treatment-related progress to both patient and therapist that are not captured through traditional pain-related questionnaires.

This study has several important limitations. First, qualitative data was only collected cross-sectionally. This means that the reported “change” is contingent on how well patients remember their pretreatment status. Future research will need to collect qualitative data prospectively, ideally using pretreatment, posttreatment, and follow-up interviews to better understand how perceptions evolve throughout the rehabilitation process. A second limitation is that only “negative” factors were measured via self-report questionnaires. While the measures included in the present study are arguably some of the most widely referenced constructs within the pain literature, it would be helpful for future research to explore whether the observed discordance would be resolved by also including quantitative measures that address positive factors. On the other hand, additional measures of negative psychological factors and clinical characteristics, such as anxiety and medication use, may have helped better characterize the sample and shed further light on the observed findings. Despite achieving data saturation, the sample size was relatively modest. Including additional participants may have provided a richer understanding of the perceptions and processes associated with rehabilitation. A further limitation is the relatively narrow time frame of the follow-up assessment. As discussed in the introduction, rehabilitation from chronic pain requires significant and long-term lifestyle changes. While participants in this sample did highlight ongoing challenges, the overarching emphasis was on personal growth. It is possible that individuals within the first six months since discharge may still be in a “honeymoon” phase of recovery and that the observed emphasis on personal development would subside with longer follow-up assessments. Future research will need to build on this work to explore this possibility.

## 5. Conclusions

Despite the limitations of this study, the findings help to advance the emerging mixed-methods literature in the area of pain as well as our understanding of patients' perceptions following chronic pain rehabilitation. Our findings show that, despite limited improvement on pain-related questionnaires following chronic pain rehabilitation, patients can experience an important and enduring sense of personal growth, which is facilitated by several patient and treatment-related factors. These findings highlight how mixed-method assessments that focus on both positive and negative factors can help provide a more comprehensive picture of how patients perceive recovery and point to the importance of developing more comprehensive models of pain-related disability. Future work will need to determine whether incorporating qualitative interviewing and clinical assessments that focus on positive factors will help facilitate further experiential learning and personal growth over the course of treatment and whether mixed-methods assessments can help advance our understanding of adherence to activity-based treatments.

## Figures and Tables

**Table 1:** Definitions of themes and subthemes derived from participant interviews.

Theme or subtheme	Definition
Personal growth	Perceived positive development in the domains of acceptance, resilience and capacity, motivation to engage in meaningful activity, and/or self-worth
Acceptance	Willingness to live with the pain condition and its related limitations.
Resilience and perceived capacity	Perceived physical and mental fitness and ability to overcome obstacles.
Motivation for meaningful activity	Openness to and engagement in valued activities.
Self-worth	The priority placed on personal needs and desires.

Factors supporting personal growth	Aspects of the person or the program that triggered or supported change, development, or progress.
Mindset entering the program	A variety of individual characteristics that participants brought to the program that support change, development, and progress.
Perceived environment of treatment	The perception that the treatment environment was both supportive and validating.
Experiential learning	The process of learning through doing in conjunction with being encouraged to reflect on one's progress.
Practical/personal tools	The recommendation of strategies that are relevant and applicable to each unique individual.

Ongoing challenges	Perceived barriers of living with chronic pain that continue to pose challenges to personal growth.

**Table 2:** Representation of personal growth subthemes among participant interviews.

Participant ID	Personal growth			
	Acceptance	Resilience and perceived capacity	Motivation for meaningful activity	Increased self-worth
INT001	X	X	X	X
INT002	X	X	X	X
INT004	X	X	X	X
INT005	X	X	X	X
INT008	X	X	X	
INT009		X	X	X
INT011		X	X	X
INT012	X	X		X
INT013	X	X	X	
INT014		X	X	
INT010		X	X	
INT003		X	X	
INT007		X		

**Table 3:** Questionnaire scores at pretreatment, posttreatment, and follow-up assessments and relation to previously established cut-scores.

ID	Multidimensional Pain Inventory Pain Severity Scale				Pain Disability Index				Pain Catastrophizing Scale			Pain Health Questionnaire-9		
	Pretreatment	Posttreatment	Follow-up	Pretreatment to follow-up percent change^a^	Pretreatment	Posttreatment	Follow-up	Pretreatment to follow-up change^b^	Pretreatment	Posttreatment	Follow-up^c^	Pretreatment	Posttreatment	Follow-up^d^
001	3.33	4.67	2.33	−**30.03** ^e^	46	36	31	−**15.00** ^e^	33	21	**11** ^e^	20	11	**9** ^e^
002	5.33	3.33	4.50	−15.57	52.50	22.50	37	−**15.5** ^e^	44	31	27	24	21	14
003	4.50	4.83	5.83	29.56	63.50	48	64	0.50	47	48	52	23	22	24
004	2.67	3.33	2.11	−20.97	18	18	23	5.00	17	18	**10** ^e^	12	13	**7** ^e^
005	4.33	1.33	1.67	−**61.43** ^e^	37	19	24	−**13.00** ^e^	30	4	**7** ^e^	16	5	**9** ^e^
007	3.67	2.33	3.33	−9.26	47	15	41	−6.00	26	12	**16**	n/a	2	**5** ^e^
008	5.33	5.00	6.00	12.57	55	50	63	8.00	41	32	30	10	11	13
009	4.33	2.00	3.67	−15.24	51	36	52	1.00	38	18	**17**	16	8	**3** ^e^
010	3.00	3.67	2.00	−**33.33** ^e^	39	48	32	−7.00	31	38	37	20	14	11
011	5.17	4.57	5.00	−3.29	46	47	45	−1.00	40	32	35	14	14	14
012	4.67	n/a	n/a	n/a	52	n/a	n/a	n/a	32	n/a	n/a	16	n/a	n/a
013	5.00	5.00	4.33	−13.40	50	37	44	−6.00	36	33	33	17	8	**9** ^e^
014	5.33	5.00	5.67	6.38	48	41	56	8.00	26	27	33	11	12	13

^a^Clinically meaningful reductions in pain severity are indicated by a 30% reduction or more.

^b^Clinically meaningful differences are indicated by a 9.5-point reduction or more.

^c^A clinically meaningful cut-off score for the Pain Catastrophizing Scale is 20.

^d^A cut-off score of 10 and above indicates major depressive disorder.

^e^Indicates that this score (in bold) satisfies criteria for cut-score/clinically meaningful change at follow-up.

n/a = participant did not complete the questionnaire. *Note*: Participant 11 did not complete the postprogram or follow-up questionnaires due to early program drop-out but did participate in the interview.
